# Case report: Gastric adenosquamous carcinoma with EBV-positive component of squamous cell carcinoma mixed with gastric carcinoma with lymphoid stroma: A novel case report and literature review

**DOI:** 10.3389/pore.2023.1610902

**Published:** 2023-02-02

**Authors:** Chang Lu, Jizhen Feng, Zhigang Yao, Lei Shi, Jiamei Li

**Affiliations:** ^1^ Department of Pathology, Shandong Provincial Hospital, Shandong University, Jinan, Shandong, China; ^2^ Department of Radiology, Shandong Provincial Hospital Affiliated to Shandong First Medical University, Jinan, Shandong, China; ^3^ Department of Pathology, Shandong Provincial Hospital Affiliated to Shandong First Medical University, Jinan, Shandong, China; ^4^ Department of Gastroenterology, Shandong Provincial Hospital Affiliated to Shandong First Medical University, Jinan, Shandong, China

**Keywords:** case report, gastric cancer, Epstein-Barr virus, adenosquamous carcinoma, gastric carcinoma with lymphoid stroma

## Abstract

**Background:** Gastric adenosquamous carcinoma with EBV-positive component of squamous cell carcinoma mixed with gastric carcinoma with lymphoid stroma are extremely unusual variants of gastric carcinoma. We herein reported such a case and summarized five related cases that have been reported previously.

**Case presentation:** A 59-year-old man was admitted to our hospital with upper abdominal discomfort and acid reflux. Gastric endoscopic examination revealed an irregular ulcer in the gastric angle. Biopsy of the lesion revealed adenocarcinoma. The patient underwent laparoscopic distal gastrectomy with lymph node dissection subsequently. Histologically, the tumor showed coexistence of GASC and GCLS. SCC and GCLS were positive for EBER *in situ* hybridization, while adenocarcinoma component was negative. Accordingly, the present case was diagnosed as GASC with EBV-positive component of SCC mixed with GCLS.

**Conclusion:** GASC with EBV-positive component of SCC mixed with GCLS is extremely rare. Although the pathogenesis of GASC and the role of EBV in the development of an ASC component have not been fully elucidated, this case will help clinicians and pathologists better understand this special subtype of gastric tumor.

## Introduction

Epstein-Barr virus-associated gastric cancer (EBVaGC) refers to a special subtype gastric cancer in which the presence of EBER expression has been confirmed by *in situ* hybridization (ISH). Generally, EBVaGC is histologically classified as adenocarcinoma (AC) and gastric carcinoma with lymphoid stroma (GCLS). GCLS is a rare type of gastric cancer characterized by intense stromal lymphocytic infiltration. Gastric adenosquamous carcinoma (GASC) is an unusual entity with distinctive characteristics. GASC mixed with GCLS associated with EBV infection is extremely rare. Only four cases (1–4) of adenosquamous carcinoma (ASC) associated with EBV have been reported so far, including three cases of GASC associated with GCLS in which EBER staining was positive only in the GCLS region and another case in which EBER staining was positive throughout all components. Besides, one study showed that EBV infection was detected in the SCC component of GCLS with focal SCC differentiation (5).

We herein reported a case of GASC with a component of SCC positive for EBER mixed with GCLS. The peculiar finding was that both squamous cell carcinoma (SCC) and GCLS components were EBER positive, whereas the AC component was EBER negative.

## Case presentation

**FIGURE 1:**
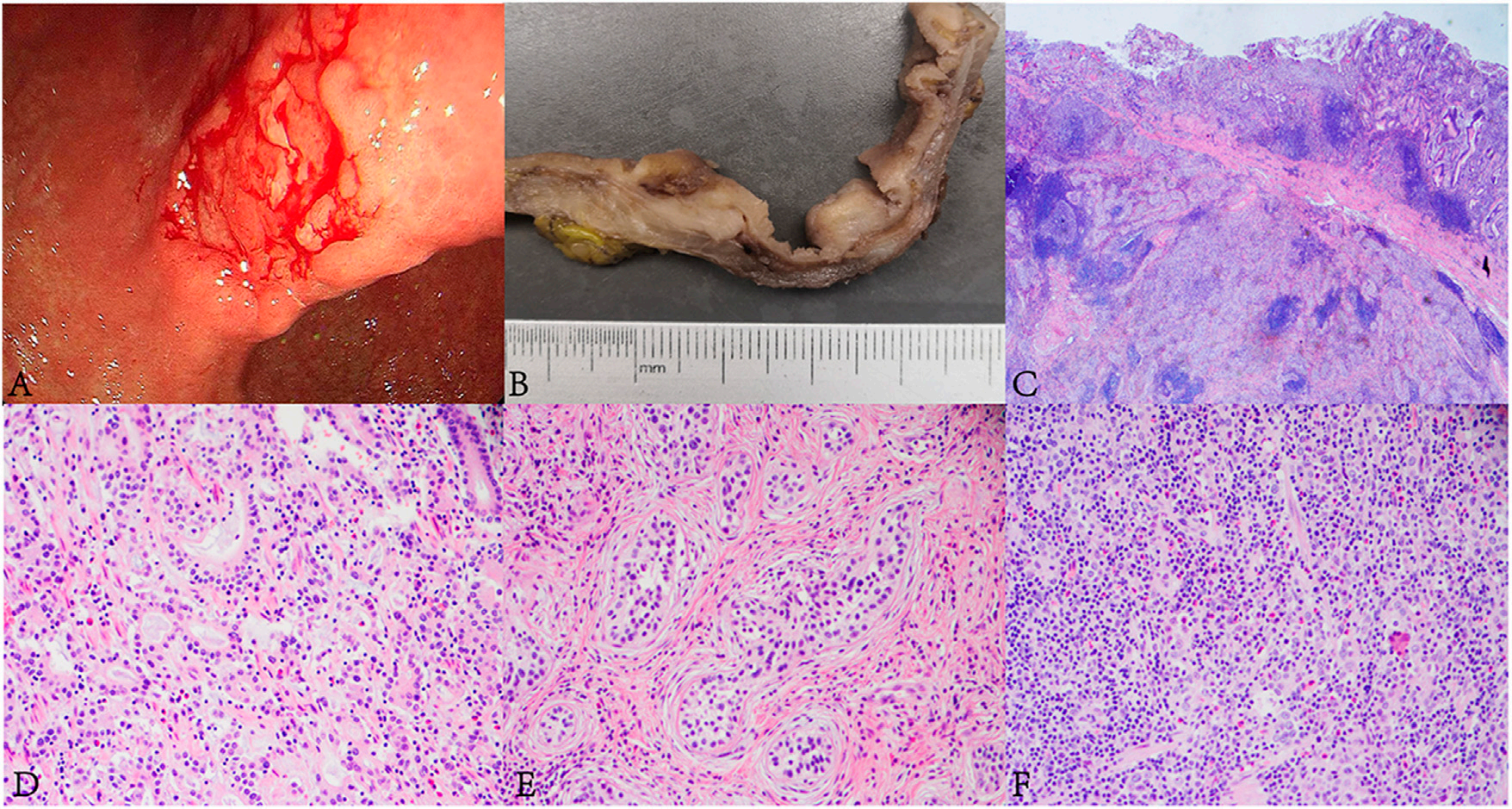
Endoscopic, and histopathological findings of the lesion. **(A)** Gastric endoscopy revealed a 2 cm × 3 cm irregular ulcerative tumor in the gastric angle. **(B)** The tumor appeared greyish white on the cut surface. **(C)** The lesion was composed of AC, SCC and GCLS components (HE, ×10). **(D)** Well differentiated AC was arranged in tubular and lace-like (HE, ×400). **(E)** SCC was arranged in nests with inconspicuous keratinization and intercellular bridges. A large number of lymphocytic infiltrations were observed in the stroma (HE, ×400). **(F)** The GCLS was characterized by irregular sheets, ill-defined tubles or single cells accompanying with dense lymphocytic infiltration (HE, ×400).

A 59-year-old man was admitted to our hospital in January 2021 due to upper abdominal discomfort and acid reflux. The patient had no significant past medical or family history. The patient’s 13C-urea breath test was negative after receiving anti-Helicobacter pylori therapy. Serum tumor biomarker examinations including CEA, CA199, CA125, CA724 and AFP were all at normal levels. Quantitative level of EBV-DNA in serum was normal. Gastric endoscopy revealed a 2 cm × 3 cm irregular ulcerative lesion in the gastric angle (Figure 1A), and biopsy of the lesion revealed adenocarcinoma. The patient subsequently underwent laparoscopic distal gastrectomy with regional lymph node dissection.

**FIGURE 2:**
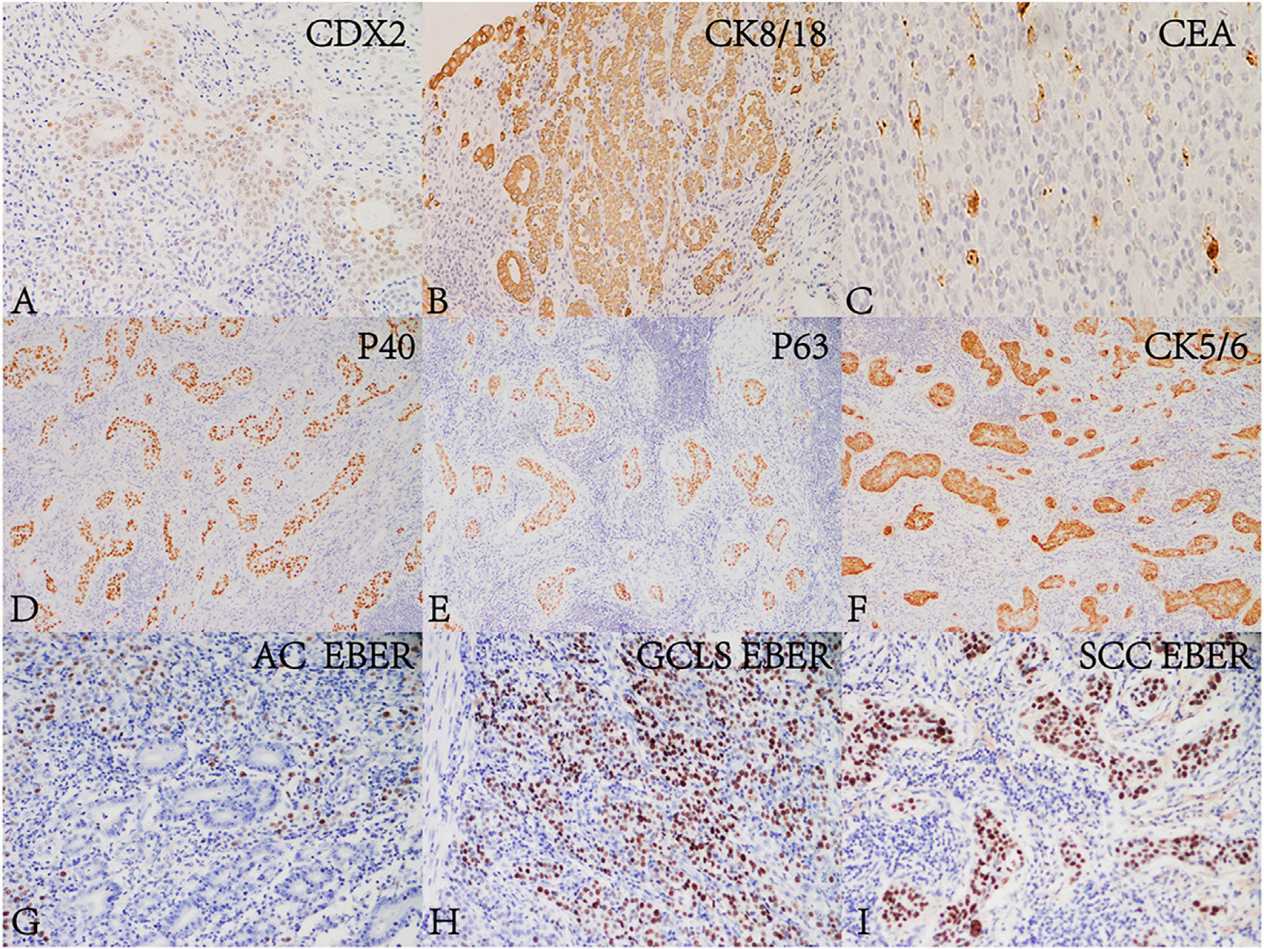
Immunohistochemical and *in situ* hybridization findings of the lesion. AC and GCLS components were positive for **(A)** CDX2 (Envision, ×200), **(B)** CK8/18 (Envision, ×200) and **(C)** CEA (Envision, ×200). SCC was positive for **(D)** P40 (Envision, ×200), **(E)** P63 (Envision, ×200) and **(F)** CK5/6 (Envision, ×200). The glandular epithelium in **(G)** AC (×200) was EBER negative. The nuclei of neoplastic cells of **(H)** SCC (×200) and **(I)** GCLS (×200) were positive for EBER.

The tumor was processed for routine histological examination, immunohistochemistry (IHC) and ISH analyses. Macroscopically, the tumor appeared greyish white on the cut surface, with a maximum diameter of 4 cm, and invaded the serosa (Figure 1B). Microscopically, the tumor consisted of a mixture of SCC (accounting for approximately 70%), well differentiated AC (accounting for approximately 20%) and GCLS (accounting for approximately 10%) (Figure 1C). AC was arranged in tubular and lace-like, mainly restricted within the mucosa and partly invaded into the submucosa (pT1b) (Figure 1D). SCC tumor cells were arranged in nests with inconspicuous keratinization and intercellular bridges (Figure 1E), and invaded into the serosa (pT4a). GCLS was characterized by irregular sheets, ill-defined tubules or single cells embedded within abundant lymphocytes. Tumor cells had weak eosinophilic cytoplasm and enlarged nuclei with prominent nucleoli and vesicular chromatin (Figure 1F). The tumor invaded into the lamina propria (pT1a). In addition, transition area between AC and GCLS was observed. Some poorly differentiated GCLS cells extended to the area of AC. And there was marked infiltration of lymphocytes and lymphoid follicles in the background of all three components. Lymphovascular, venous and nerve invasion were not observed. Moreover, no metastasis was found in the lymph nodes of the greater and lesser curvature of the stomach. Immunohistochemically, AC and GCLS components were positive for CDX2, CK8/18 and CEA (Figures 2A–C), whereas SCC was diffusely positive for P40, P63 and CK5/6 (Figures 2D, E). Immunohistochemical score for HER2 expression was 1+ and mismatch repair proteins such as MLH1, PMS2, MSH2 and MSH6 remained intact. PDL1 (22C3) was negative both in tumor and inflammatory cells. Neuroendocrine markers were negative in all components. The ISH results revealed that the nuclei of neoplastic cells of SCC and GCLS were diffusely EBER positive, whereas AC was EBER negative (Figures 2G–I). Accordingly, the present case was diagnosed as GASC with EBV-positive component of SCC mixed with GCLS (pT4aN0). The patient was uneventful after surgery and underwent six cycles of chemotherapy with oxaliplatin (200 mg dL) combined with tegafur (60 mg/time, twice a day). The patient has been free of tumor recurrence during 18 months of medical follow-up.

## Discussion

EBVaGC is a kind of cancer with special clinicopathological features, biological behavior and prognostic characteristics, which was first defined by Tokunaga et al. (6) in 1993. In 2014, The Cancer Genome Atlas (TCGA) (7) listed EBVaGC as an independent molecular subtype of gastric cancer. The characteristic of EBVaGC has been revealed, it accounts for nearly 10% of gastric carcinoma worldwide with variable frequencies between geographic regions. EBVaGC is more common in males and young adults, and preferentially occurs in the proximal stomach and the remnant stomach. The typical gross feature is marked by superficial, indurated, ulcerated, or saucer-shaped tumors, as well as a significant thickening of the tumorous wall (8).

ASC, which was first reported by Rolleston in 1905, consists of both AC and SCC components, with SCC accounting for more than 25% of the tumor. GASC is pretty rare and accounts for less than 1% of gastric cancers (9). Compared to the traditional gastric AC, GASC is more common in young males and is often diagnosed in the T4 stage. Lymphovascular, venous and nerve invasion (10) are often seen. Patients with GASC are prone to be accompanied by distant metastasis and lymph node metastasis. Distant metastasis is more common in the liver (11, 12) and the lymph node metastasis rate is about 86.4% (1). The high rate of lymph node metastasis suggests that GASC is more aggressive than other types of gastric cancers and has a poor prognosis.

Kuroda et al. (1) first reported a case of GCLS with ASC which was accompanied by EBV infection in 2010. Since then, five other relevant cases have been reported successively. Table 1 summarized the clinicopathological features of the five previously reported cases of concomitant ASC and GCLS, as well as the present one. In these cases, all patients were males with a median age of 58.5 (range 50**–**67) years, and the majority of them sought medical attention for stomach pains. Lymph node metastases from all three components were observed, with AC being the most common component. In the reported cases, EBER staining was only positive in the GCLS region in three cases (1-3), while it was throughout positive in one case (4). In the Ji-Hoon’s report (5), EBER staining was diffusely positive in GCLS and focally positive in SCC. To the best of our knowledge, our patient is the first case in which EBER staining was negative in the AC part but diffuse positivity was detected in SCC and GCLS components.

**TABLE 1:** Clinicopathological features of Epstein-Barr virus-associated gastric adenosquamous carcinoma.

Authors (publication year)	Age/sex	Location	Size (cm)	EBER	Lymph node metastasis	Invasion depth	Prognosis
Kuroda (2010) (1)	67/M	Upper	6 × 5	AC (−)	NA	NA	NA
				SCC(−)			
				GCLS (+)			
Oya H (2010) (2)	50/M	Angular	9 × 8.5	AC (−)	NA	NA	AWD in 10 years
				SCC(−)			
				GCLS (+)			
Miyake (2019) (3)	58/M	Antrum	Two lesions	AC (−)	5/39 (ASC/GCLS)	ASC (pT4a)	AWD in 8 months
			GASC:3.5 × 2.5	SCC (−)		GCLS (pT2)	
			GCLS:4.5 × 3.6	GCLS (+)			
Kim Ji-Hoon (2019) (5)	58/M	Angle and body	Two lesions	SCC (focal +)	8/39 (GCLS)	GCLS with focal SCC (pT1b)	Recurrence in 12 months and death in 25 months
			GCLS with focal SCC: 1.1 × 1.1GCLS:3.0 × 2.0	GCLS (+)		GCLS (pT1b)	
Cao (2022) (4)	58/M	Body	3 × 3	AC (+)	1/54 (AC)	AC (pT1b)	AWD in 8 months
				SCC (+)		SCC (pT1b)	
				GCLS (+)		GCLS (pT1b)	
Present case	59/M	Angular	3 × 2	AC (−)	0/23	AC (pT1b)	AWD in 18 months
				SCC (+)		SCC (pT4a)	
				GCLS (+)		GCLS (pT1a)	

M, male; AC, adenocarcinoma; SCC, squamous cell carcinoma; ASC, adenosquamous carcinoma; GASC, gastric adenosquamous carcinoma; GCLS, gastric carcinoma with lymphoid stroma; AWD, alive without disease; EBER, Epstein-Barr virus encoding RNA.

ASC mixed with GCLS associated with EBV infection is very rare and its pathogenesis has not been clearly defined. Kuroda et al. (1) had proposed two possibilities. One was the two tumors collided and seemed to grow into a mass. The other was AC differentiated into two forms: one showed differentiation toward EBV-related tumors and the other demonstrated squamous differentiation. A mixture of AC and SCC can be seen in four cases, and even a transition of AC and SCC has been observed (4). In addition, AC is the most common component of the metastatic lymph nodes of patients with gastric ASC (3, 4, 13). In the present case, considering the presence of a transition area between AC and the other two components with EBER positive expression, we suggest the second possibility.

EBVaGC has unique genetic and epigenetic features, which could be potential targets for cancer treatment. It is noteworthy that EBVaGC is associated with multiple gene alterations such as PIK3CA, CTNNB1 and ARID1A. Abundant lymphocytes infiltrating around and inside the tumor are highly expressed PD-L1/PD-L2, suggesting patients with EBVaGC may benefit from immunotherapy. PD-L1 expression was detected in three cases including ours. In one case (5), PD-L1 was focally expressed in approximately 5% of SCC cells. And in another case (4), combined positive score for PD-L1 expression was 15, mainly positive expression on lymphocytes. However, PD-L1 expression was negative in the present case. Four of the cases, including ours, received chemotherapy after surgery.

EBVaGC usually has a superior biological behavior and prognosis than other types of gastric cancer. However, GASC is more aggressive and has an inferior prognosis compared to conventional gastric AC, even including signet ring cell carcinoma (14). Therefore, the prognosis of GSAC with partial EBV-positive component remains unknown and is worthy of further exploration. Oya H et al. (2) observed the longest disease-free survival period, which was 10 years, indicating the importance of long-term surveillance in patients with EBVaGC, particularly in patients with lymph node metastases. Additionally, abdominal/pelvic multiphasic CT or abdominal/pelvic MR, physical examination, and biochemical tests should be performed during the follow-up.

In summary, we reported here the first case of GASC with EBV-positive SCC mixed with GCLS, characterized by diffuse EBER positivity in both the SCC and GCLS components, but not in the AC part. Our case may help clinicians and pathologists to better recognize and pay attention to this special subtype of gastric cancer. Further researches are needed to explore the pathogenesis, treatment and biological behavior of this type of tumor.

## Data Availability

The original contributions presented in the study are included in the article/supplementary material, further inquiries can be directed to the corresponding authors.
